# Metastability and Inter-Band Frequency Modulation in Networks of Oscillating Spiking Neuron Populations

**DOI:** 10.1371/journal.pone.0062234

**Published:** 2013-04-16

**Authors:** David Bhowmik, Murray Shanahan

**Affiliations:** Department of Computing, Imperial College London, London, United Kingdom; University of British Columbia, Canada

## Abstract

Groups of neurons firing synchronously are hypothesized to underlie many cognitive functions such as attention, associative learning, memory, and sensory selection. Recent theories suggest that transient periods of synchronization and desynchronization provide a mechanism for dynamically integrating and forming coalitions of functionally related neural areas, and that at these times conditions are optimal for information transfer. Oscillating neural populations display a great amount of spectral complexity, with several rhythms temporally coexisting in different structures and interacting with each other. This paper explores inter-band frequency modulation between neural oscillators using models of quadratic integrate-and-fire neurons and Hodgkin-Huxley neurons. We vary the structural connectivity in a network of neural oscillators, assess the spectral complexity, and correlate the inter-band frequency modulation. We contrast this correlation against measures of metastable coalition entropy and synchrony. Our results show that oscillations in different neural populations modulate each other so as to change frequency, and that the interaction of these fluctuating frequencies in the network as a whole is able to drive different neural populations towards episodes of synchrony. Further to this, we locate an area in the connectivity space in which the system directs itself in this way so as to explore a large repertoire of synchronous coalitions. We suggest that such dynamics facilitate versatile exploration, integration, and communication between functionally related neural areas, and thereby supports sophisticated cognitive processing in the brain.

## Introduction

There has been growing interest in brain dynamics and oscillatory behaviour within neuroscience communities due to the realization that different perceptual and behavioural states are associated with different brain rhythms. The oscillatory activity of large populations of neurons observed in the local field potential (LFP) can be the result of complex dynamics at a number of scales: from a role played in theta LFP by subthreshold membrane potential oscillations of individual neurons [1], to population entrainment by the rhythmic firing of pacemaker neurons [2], [3], as well as re-entrant architectures involving inhibitory interneurons that result in LFP gamma oscillations [3]. As Buszaki and Draguhn claim: *‘The synchronous activity of oscillating networks is now viewed as the critical “middle ground” linking single-neuron activity to behaviour*’ [4].

The various rhythms have diverse associations. Thalamocortical networks display increased delta band (0.1−3.5 Hz) power during deep sleep [5]. Theta (4−7.5 Hz) activity is increased during memory encoding and retrieval [6]. Alpha band (8−13 Hz) changes are associated with attentional demands [7]. Beta (14−30 Hz) oscillations have been related to the sensorimotor system [8]. Of all the frequency bands the role of gamma (30–80 Hz) is thought to be most extensive and is hypothesized to provide a mechanism that underlies many cognitive functions such as: attention [9], associative learning [10], working memory [11], the formation of episodic memory [12], [3], visual perception [13], and sensory selection [14].

The evidence suggests that basic modes of dynamical organization are reflected in brain rhythms [15]. In addition the “communication through coherence” hypothesis proposes that such synchronization opens up communication channels between distant neuronal groups[16], providing optimal conditions for information transfer [17]. With these insights in mind it has also been suggested that transient periods of synchronization and desynchronization provide a mechanism for dynamically integrating and forming coalitions of functionally related neural areas [18].

Such transient dynamics have been demonstrated in systems of phase lagged, delayed and pulse coupled oscillators that have been organized into a modular community structured small world networks akin to those found in the brain [18], [19]. These systems exhibit interesting phenomena such as: metastability, chimera-like states and coalition entropy. Metastability is quantified by the variance of synchrony within an individual oscillator cluster over time, averaged for all clusters in the system, and so characterizes the tendency of a system to continuously migrate between a variety of synchronous states. Fixing time and calculating the variance across clusters gives an index of how chimera-like the system is, indicating the level of spontaneous partitioning into synchronized and desynchronized subsets. Coalition entropy measures the variety of metastable states entered by a system of oscillators and is calculated from the number of distinct states the system can generate and the probability of each state occurring. As a collection these measures capture the ability and tendency of a system to best explore the space of dynamic synchronous coalitions. In the afore-mentioned work in which these transient dynamics were demonstrated, a key area within the oscillator network parameter space was identified where the combination of these measures is optimal. An embodied neural oscillator system tuned to such a sweet spot would facilitate versatile exploration, integration and communication of functionally related areas throughout the behavioural problem solving process.

It is increasingly common for simple oscillator models to be used as abstractions of oscillating neural populations in brain modelling [20]. Whilst there is a greater perceived affinity to neural systems when moving from phase lagged, to delayed, to pulse coupled oscillator system, our previous work experimentally demonstrated that such oscillator models display close behavioural similarities to networks of oscillating neural populations [21]. However, the latter work illustrates how neural models display greater spectral complexity during synchronization than more abstract oscillator models, with several oscillatory frequencies coexisting within an individual neural oscillator population. This work explored the relationship between simple oscillator models and their neural population cousins by emulating neurally the Kuramoto critical coupling experiment [22] which showed an increase in synchrony as connection strength is increased in a uniformly connected network of simple oscillators. It was demonstrated that at the point of maximum synchrony the neural systems not only displayed several coexisting frequencies within an individual oscillator population but that the system also showed deviations from a measure of full synchrony likely caused by these additional fluctuating influences.

The spectral complexity of neural systems has been observed *in vivo* [23]. It has been hypothesized that slower oscillations provide a framework for other faster oscillations to operate such that fast oscillations communicate content while slow oscillations mediate transient connectivity [3]. Very large networks are recruited during slow oscillations whereas higher frequency oscillations are confined to a small neuronal space [4]. Widespread slow oscillations modulate faster local events. Some such interactions have received much attention, for example the nesting of gamma in theta during memory formation [24], [25]. However, the phenomenon as a whole is not well understood. Within the same neuronal structure neighbouring frequency bands, which are typically associated with different brain states, coexist but compete with each other. However, several rhythms temporally coexist not only in the same structure but also in different structures and interact with each other [4]. How these different frequencies affect each other across populations is an area demanding much exploration and is the focus of this paper.

Much research has focused on measuring the effect when different populations of neurons synchronize to the same frequency [17], [26], [27], with further interest in correlations across frequency bands, as for example assessed by the mean local time-frequency energy correlation [28]. It has been shown that, within a single neural population coexisting oscillatory frequencies in different bands start, stop and restart. Further to this we show that these frequencies fluctuate. The frequency of an oscillating population does not remain at a constant but may speed up and slow down over time. The aim of this work is to understand how the fluctuation in the frequency of one neural populations'oscillation over time affects the other neural populations it is connected to. The results in this paper demonstrate that the fluctuation in frequency in one neural population modulates the fluctuation in frequency in other neural populations, and that this influence increases with greater structural connectivity between the populations. Due to the connective interdependency of each population to the others in a network, the fluctuating oscillatory frequency of each population modulates the other populations' oscillatory frequencies. It is shown that, this interaction of fluctuating frequencies in the network as a whole is able to drive different populations towards episodes of synchrony.

The approach taken in this paper is to build simulations of interacting neural oscillator populations, to capture in detail the intermittent fluctuating frequencies in each oscillator as fragments of times series (time series *strands*), and to correlate these strands against other such strands across bands and across neural populations. We average this correlation measure for the network as a whole in order to give a *mean intermittent frequency correlation*. This is then contrasted against measures of synchrony and coalition entropy in the network as a whole. By varying the amount of structural connectivity between neural populations we show that the interaction of fluctuating frequencies in different bands and across neural populations directly relates to synchrony, and that this correlation measure is inversely related to coalition entropy in the network. Further to this, we identify an area in the connection space at which the causal interaction of fluctuating frequencies across neural populations and the coalition entropy of the system are optimal. The latter entails that the fluctuating frequencies in different populations are not only modulating each other so as to drive each other towards episodes of inter-population synchrony, but also that the variation in the make-up of these synchronous coalitions over time is very high. We hypothesise that such dynamics would form a good basis for contextual exploration, as well as integration among, and communication between functionally related areas during cognitive processing.

The paper is organized as follows. First we describe the neural models used in our experiments. After this we describe methods for generating neural oscillator architectures using a genetic algorithm. Following this, the method for extracting intermittent frequency strands from each oscillator is detailed before explaining how these are used to obtain a measure of mean intermittent frequency correlation for the network. The measures for synchrony and coalition entropy are then detailed. The experiments and results follow this and we close with a brief discussion.

## Methods

### Neural models

Hodgkin [29] distinguishes between types of neuron responses. The first type of neuron (Type I) always responds to small depolarization by advancing the next spike. An example of such a neuron is the integrate-and-fire model. The second type (Type II) is exemplified by the Hodgkin-Huxley model in which there is a negative region just after the refractory period, where a depolarization delays the firing of the next spike because the delayed rectifier potassium current is greater than the sodium current, while an excitatory post-synaptic potential received at a later time advances the firing. In this paper both Type I and Type II models are assessed.

### Quadratic integrate-and-fire neurons

The QIF model [30] displays Type I neuron dynamics [31]. The time evolution of the neuron membrane potential is given by:
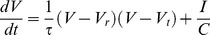
where *V* is the membrane potential, with *V_r_* and *V_t_* being the resting and threshold values respectively. *C i*s the capacitance of the cell membrane. *τ* is the membrane time constant such that *τ* = *RC* with *R* being the resistance. *I* represents a depolarizing input current to the neuron.

An action potential occurs when *V* reaches a value *V_peak_* at which point it is reset to value *V_reset_*. The QIF model is equivalent to the theta neuron model described by Ermentrout and Kopell [32] if one sets the reset condition *V_peak_* = ∞ and *V_reset_* = −∞. Like Börgers and Kopell [33] we use values *V_r_* = *V_reset_*_ = _0 and *V_t_ = V_peak_* = 1, which reduces the above equation to:
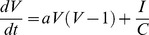



Here 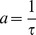 and is set to the value 2 for all experiments carried out in the paper. When working with the QIF model we assume a membrane potential between *V_r_* = −65 mV and *V_t_* = −45 mV.

### Hodgkin-Huxley neurons

The Hodgkin-Huxley [34] model is widely considered as the benchmark standard for neural models. It is based upon experiments on the giant axon of the squid. Hodgkin and Huxley found three different types of ion current: sodium (*Na^+^*), potassium (*K^+^*), and a leak current that consists mainly of chloride (*Cl^−^*) ions. Different voltage-dependent ion channels control the flow of ions through the cell membrane. From their experiments, Hodgkin and Huxley formulated the following equation defining the time evolution of the model:
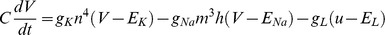


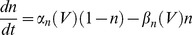


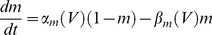


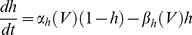



*C* is the capacitance and *n*, *m* and *h* describe the voltage dependent opening and closing dynamics of the ion channels. The maximum conductances of each channel are: *g_k_* = 120, *g_Na_* = 36 and *g_L_*  = 0.3. The reversal potentials are set so that that *E_k_* = −12, *E_Na_* = 115 and *E_L_* = 10.6. The rate functions for each channel are:
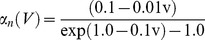


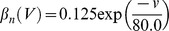


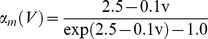


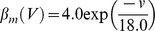


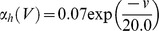


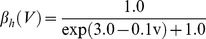



All work in this paper using the HH model adjusts the neuron resting potential from 0 mV of the standard HH implementation to the more accepted value of 65 mV [35].

### Synaptic model

The synaptic model for simulations using the QIF model is a current synapse that simply multiplies the incoming spike by a synaptic weight:
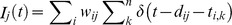
where *I_j_(t)* is the input to neuron *j* and time *t*. *w_ij_* is the synaptic weight from neuron *i* to neuron *j*, and *d_ij_* is the synaptic delay from neuron *i* to neuron *j*. A list of the all *n* spikes produced from neuron *i* during a simulation are denoted by their spike times *t_i,k_*, where *k = 1,2…..n*. δ is a delta function applied to *t-d_ij_-t_i,k_*, such that adjusting the current time *t* by the synaptic delay *d_ij_* identifies the spike production time at neuron *i* for which a spike is due to arrive at neuron *j* at time *t*. If *t_i,k_* matches this spike time then the delta function produces an output value 1.

The HH model uses conductance synapses, and so uses reversal potentials to further scale incoming spikes. The latter model is as follows:
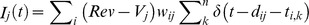



The additions to the previous synaptic model are, *Rev* which is the reversal potential, and *V_j_*, which is the voltage of the target neuron. The reversal potentials for the model are set to the same values in all experiments. For excitatory inputs the reversal potential is set to 0 mV, and for inhibitory inputs the reversal potential is −70 mV. Not using a synaptic reversal model for the QIF model is equivalent to using a synaptic reversal model with reversal potentials set to +∞mV for excitatory neurons and -∞mV for inhibitory neurons.

### Evolution of the architecture for neural oscillatory nodes

Groups of neurons firing together rhythmically can occur because of intrinsic firing patterns of excitatory principal cells or due to common input from a pacemaker, however, it is more common both in the cortex and the hippocampus that rhythmic firing happens as an emergent property of interactions between excitatory principal cells and inhibitory interneurons. Variations of this mechanism, known as pyramidal inter-neuronal gamma (PING), can give rise to both faster gamma oscillations as well as slower oscillations such as theta in the cortex and the hippocampus [3].

Excitatory neurons drive the entire local network, including inhibitory interneurons. The most strongly driven inhibitory neurons will fire first and provide inhibition to numerous other inhibitory neurons. The inhibitory effect on all these neurons will disappear at approximately the same time. Affected inhibitory neurons will then fire roughly together, causing large numbers of inhibitory neurons to be entrained to a rhythm within just a few oscillatory cycles [36]. This rhythmically synchronized inhibition also affects the network's excitatory neurons with a fast and strong synaptic input [37] thus leaving only a short window for the excitatory neurons to fire after one period of inhibition wears off and before the next one starts [38].

**Figure 1:**
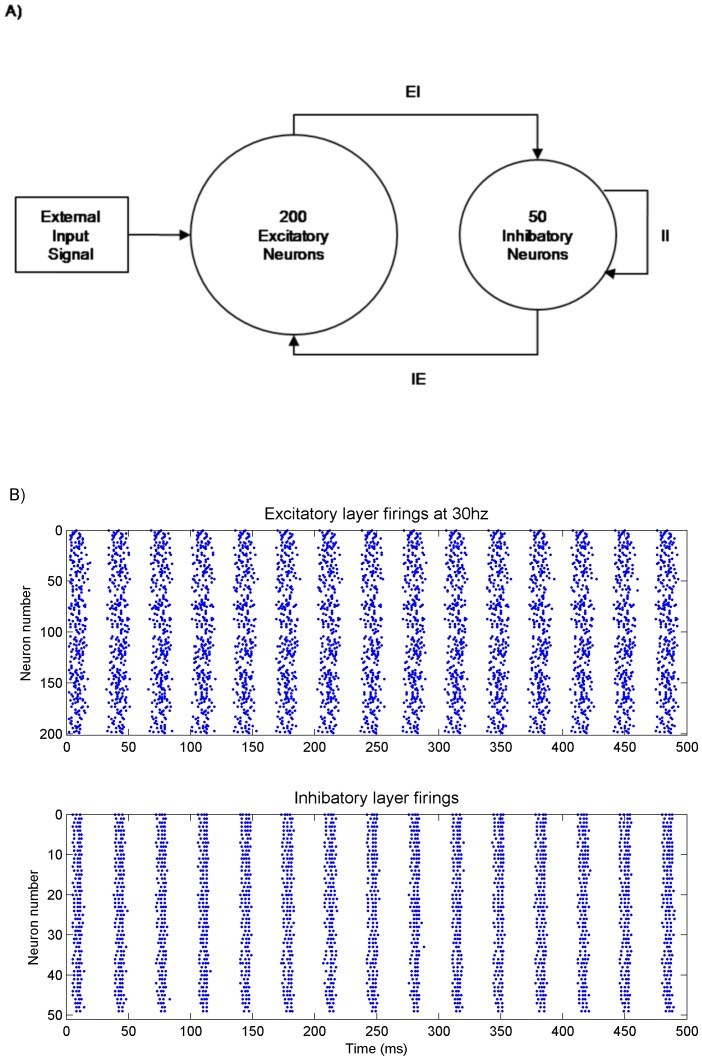
PING oscillatory architecture and behaviour. (A) The pyramidal inter-neuronal gamma (PING) architecture used for the neural oscillator nodes in the simulation experiments. To generate oscillator nodes of different frequencies for different neural models this base architecture was used with a genetic algorithm evolving the weights and delays for the synaptic connections. (B) Example of the firing behaviour of an evolved QIF PING node oscillating at 30 Hz.

The oscillators used in this work conform to a PING architecture. Whilst the general PING architecture is well understood, the specific details required for both particular oscillatory frequencies and neuron model vary and involve a large space of parameter values within the general PING framework. In order to provide a wide range of different intrinsic oscillatory frequencies for the neural oscillator nodes used in the experiment, it was decided to obtain these parameter values by use of a genetic algorithm (described below). The genetic algorithm evolved, within biologically plausible bounds, every oscillatory frequency between 30 Hz and 50 Hz for both QIF and HH models. The evolutionary mechanisms were constrained so that each neural network was evolved in accordance with the general PING architecture mentioned above. All neural populations for the PING oscillators used an excitatory layer of 200 neurons and an inhibitory layer of 50 neurons. The excitatory layer drives the entire network and so is the only one to receive external input. The input is generated from a Poisson process with parameter λ = 4.375. For QIF models the inputs were scaled by 8 and for the HH models the inputs were scaled by 15 in order to provide sufficient stimulus to induce firing. The networks were wired up with connections between inhibitory neurons (II), from excitatory to inhibitory neurons (EI) and from inhibitory to excitatory neurons (IE). Excitatory to excitatory (EE) connections were excluded in order to limit saturation effects (by which we mean all neurons firing all of the time). Saturation effects tend to arise in the later simulations in which many neural PING nodes were wired together. The possibility of saturation was not otherwise catered for in the evolutionary process due to the PING networks being evolved in isolation. The PING architecture used is illustrated in figure 1a. In addition to the synaptic weight, a scaling factor of 5 was used on all synaptic current in the oscillatory populations for both QIF and HH models to simulate networks of a larger size than we could feasibly simulate, given the number individuals in a population and the number of generations in an evolutionary run, as well as the large number of simulation runs using 10 neural PING nodes in our final experiment.

A genetic algorithm is a blind search and optimisation technique based upon the theory of natural selection [39]. Parameters are encoded in a pseudo genome, and are used to instantiate an individual, in this case a neural network. A population of individuals are tested and scored for their fitness at performing the test. Based upon their fitness ranking pairs of individuals are chosen to produce offspring for the next generation via crossover of their genomes. Mutation is then applied to some parameters in the new offspring genome. As this process continues over generations individuals in the population become optimised at performing the target task. The parameters that were evolved in this work were the synaptic weights and delays. Both of these were generated during genome expression of each individual in each generation using an approximately normal distribution, with the means and variances for the weights and the delays being the parameters in the genome evolved. The distribution is approximately normal as the weights were bound to evolve values between 0 and 1 for excitatory connections and 0 and −1 for inhibitory connections. Delays were similarly bound. Long delays are quite unrealistic for a cluster of neurons in which all neurons are anatomically close together. In the cortex synaptic latency ranges from 0.2 ms to 6 ms [40]. In order to produce realistic results, excitatory delays were bounded between 1 ms and 10 ms. The IE and II delays were allowed to have a maximum value of 50 ms to simulate the effect of slow inhibitory interneurons, the behaviour of which was otherwise not modelled.

Individuals were tested for 5000 ms of simulated time in which they received external input to the excitatory layer as described above. The fitness function for the genetic algorithm consisted first of taking the spike firing times of the excitatory population and converting it to a continuous time-varying signal. This was achieved by binning the spikes over time, and then passing a Gaussian smoothing filter over the binned data. Next a Fourier transform was performed on the mean centred signal to produce the frequency spectrum of the signal. The main fitness term was calculated by creating a scaled Gaussian centred around the desired frequency *f* in the spectrum of the form:
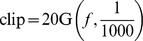



The frequency spectrum s was subtracted from this and normalized:
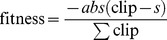



An extra penalty term was introduced to discourage frequencies outside the desired range. This was achieved by multiplying the frequency spectrum by −0.002 in the areas further away from the desired frequency whilst ignoring the area at and immediately around the desired frequency. The result was then normalized and added to the main fitness term.

The evolutionary population consisted of 20 individual genomes. For each generation, each individual was tested for 5000 ms of simulated time. After this each individual was rated for fitness and probabilistically selected for the next generations' parents based upon their fitness ranking. Crossover was performed on parent genomes after which mutation was applied to the offspring with a probability of 0.1.

All evolved weights for QIF solutions had very high means and small variances, whereas the HH solution showed greater variation in the weight means across evolved solutions for different frequencies, indicating greater sensitivity in the model and solution in that they require a very specific balance of the parameters for each particular solution. The means for the delays evolved for both QIF and HH solutions had a similar form, from which can be concluded that the EI mean delay+IE mean delay≈1000/2f. Figure 1b shows a raster plot of an evolved PING oscillator with regular bursts of firing in the excitatory layer at 30 Hz.

### Extraction of intermittent frequency strands

The work presented in this paper aims at assessing the correlation between the fluctuating frequencies in different neural oscillators that are connected together in a network. In order to achieve this we first need to extract the instantaneous frequency responses for each neural oscillator at each moment in time during a simulation. The standard techniques for doing this are to either use a short-time Fourier transform or a wavelet transform. To perform either first requires converting the firings of an oscillatory neural population into a continuous time signal upon which one of these transforms can be performed. We only use the excitatory layer in a neural oscillator when producing this signal. The signal is obtained by first binning the number of spikes at each moment in time for the excitatory layer, and then passing a Gaussian smoothing filter over the data. Finally the signal is centred around its mean to obtain the continuous time signal upon which we can perform the transform.

**Figure 2:**
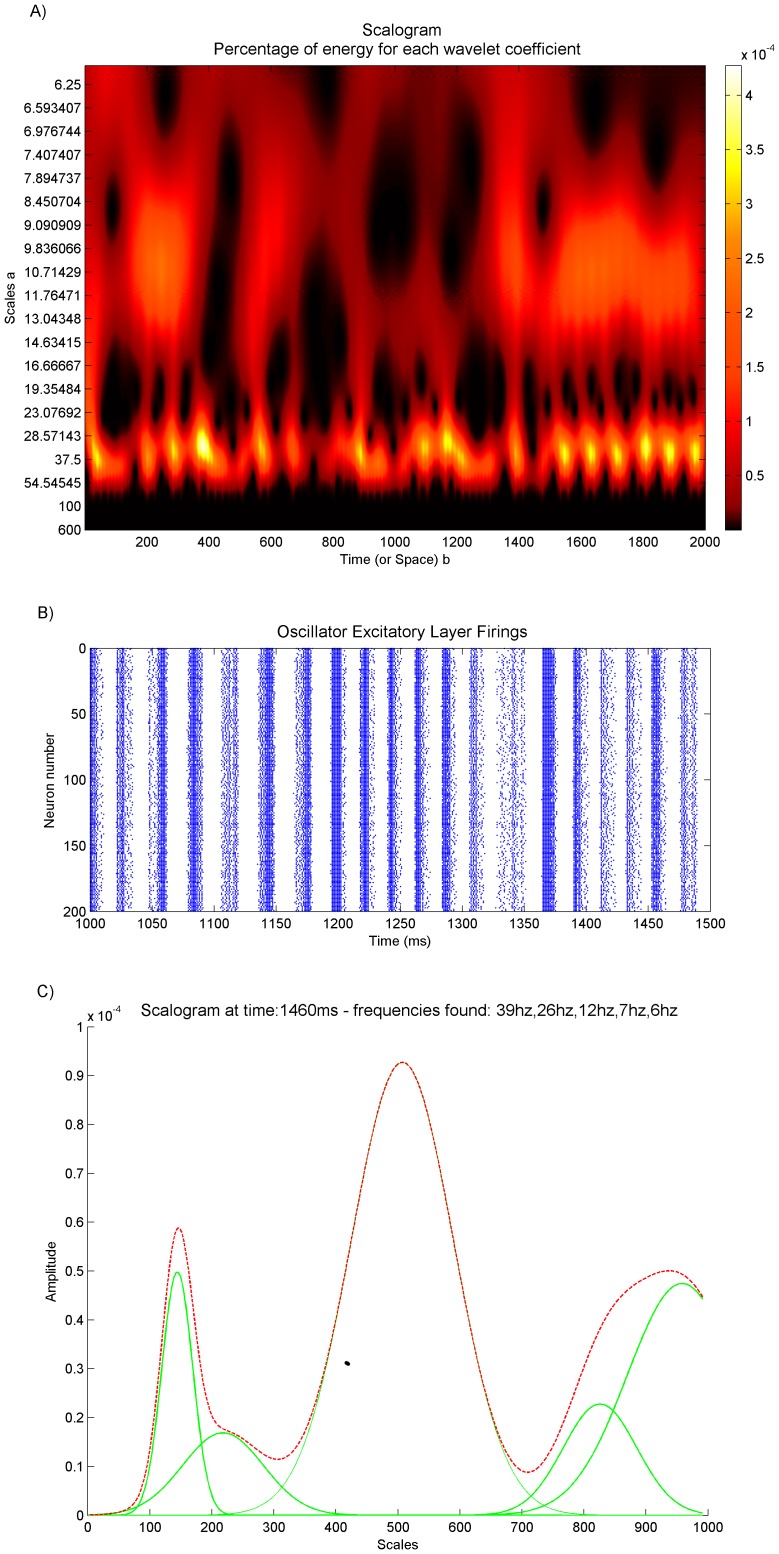
Extraction of frequencies from population firings (A) Scalogram of the excitatory layer of a neural PING node that has been connected to 9 other nodes each oscillating at a different frequency. (B) Firing behaviour of the same excitatory layer between 1000 ms to 1500 ms in the simulation. Note how the spacing in between the burst of firing is reflected as different frequencies in the scalogram in panel A. (C) Time slice of the scalogram in panel A taken at 1460 ms. The red line shows the time slice and the green lines show different Gaussians, the sum of which fits the red line.

Both Fourier and wavelet based approaches for extracting the time-frequency information from a signal suffer from shortcomings due to the time-frequency uncertainty principle. A Gabor wavelet transform has been chosen for use in this work, because the responses of Gabor wavelets have optimal properties with respect to the time-frequency uncertainty principle [41]. The Gabor wavelet used had a centre frequency of 0.6 Hz and was applied with a continuous wavelet transform using scales from 1 to 100 in increments of 0.1, and a delta of 0.001. Figure 2a shows the scalogram of a wavelet transform taken from the excitatory layer of a neural oscillator in one of the experiments. In this experiment, as in all others, the oscillator was placed in a network with 9 other oscillators, each oscillating at a different intrinsic frequency. There is a given probability of connecting each oscillator to another in the network, and a given synaptic connection weight for the connections formed between oscillators. Both connection probability and synaptic weight are unique to each experimental run. Figure 2b shows a raster plot of the firing behaviour of the same excitatory layer between time points 1000 ms and 1500 ms in the simulation. It can be seen that the spacing of the bursts of firing between 1050 ms and 1150 ms is wider and thus at a different slower frequency to the spacing of the bursts of firing between 1200 ms and 1275 ms. The wavelet response to the slow and then faster bursting can be seen as a difference from low to high frequency on the scalogram in the same temporal area and around the frequency range from 30 Hz–50 Hz. These responses are deviations from the regular 33 Hz bursting that the PING oscillator was evolved to fire at and are due to the interaction with the other oscillator nodes.

The Gabor wavelet produces a blurred impulse response around given frequency responses at each point in time. The blurring from a Gabor wavelet is in the form of a Gaussian [41], as illustrated by a time slice at time point 1460 ms shown in figure 2c taken from the scalogram in figure 2a. Further techniques have to be applied to the transformed data in order to extract the instantaneous frequency information. Standard ridge and skeleton methods do not perform well when there are many components, some of which remain very close for a while and separate again, or when they can die out, or when new ones can appear from nowhere [42]. As can be seen by the scalogram in figure 2a the data in the work presented here is of this type. Drawing upon the Gaussian nature of the impulse response from the Gabor wavelet, we apply a technique of fitting a sum-of-Gaussians model to the transformed data at each point in time [41]. Figure 2c shows such a fitting. Identifying the means and magnitudes of the means of the fitted Gaussians gives the instantaneous frequencies and their amplitudes respectively.

The next stage in preprocessing the data requires forming a time series of the instantaneous frequencies as they fluctuate over time, what we call a *strand*. These fluctuating frequency responses may also be intermittent due to the frequency response dropping out and starting again. Zero values are substituted into the time series strands during the drop out moments to identify the fact that there is no frequency response at those times. There may be many coexisting frequencies for each neural oscillator at each time point in a simulation, and therefore many coexisting strands. To obtain these strands, after the instantaneous frequencies at each point in time for an oscillator are calculated, the movement of each frequency is tracked over time so as to link them together into a single time series fragment.

The algorithm for forming the frequency time series strands has three parts. The first part is simply to sequence the nearest frequencies in time into a strand as follows:

T = start_time.

**while** T is not equal to end_time.

 For each unassigned instantaneous frequency at time point T create a new strand containing that frequency.

 T = T+1.

**while** there are strands and frequencies within the distance limit L.

 Find the strand at time point T-1 with the closest frequency to one of the instantaneous frequencies at time point T.

 If the frequency is within limit L add the frequency to the strand and remove the strand from further consideration until the next iteration.

**end**

**end**

Further to this, we need to cope with bifurcations in the oscillator behaviour when an oscillator in a particular state A1, flips to another state B1, and then flips again to a state A2 such that states A1 and A2 have the same number of coexisting frequencies and these frequencies have approximately the same values. Hence the system is returning to its original state (A1) after the middle state B1. In each state there may be several coexistent frequency strands. We wish the strands in the original state A1 and its return state A2 after the middle state, to be stitched together so as to maximize the strand length and as a result the correlation. The distance between the frequencies in the strands in the state A1 and state A2 may be near enough within a limit L to make a direct match as in the previous algorithm, due to the fact that there is a close continuation between the two. However, there are situations in which the values of the frequencies in A2 are not near enough for direct matching, but instead have values similar to how state A1 would have been at that time if the bifurcations had not occurred and state A1 had instead continued developing. That is to say, whilst being the same state as A1, state A2 is in a later stage of development. In such a situation we use regression to project where the frequencies of strands in the original state A1 would have progressed to, and match these projections to the frequencies of the strands in A2. We apply a maximum frequency distance limit L as before on this projected matching.

In order to stitch states in this way, we first group all strands together which share the same start time so as to identify them as being in the same state. The state matching algorithm then preferentially matches states nearest to each other whose strands have the closest frequencies or projected frequencies. There is a maximum time limit between states for which we allow such stitching to occur. In order to get the best matching between states, we first perform the algorithm with the constraint that stitched states must contain the same number of strands, and then perform the algorithm again without this constraint.

After the state stitching has been carried out we extract the individual strands that are contained within the states, as we only consider pairs of individual strands during correlation. The strands are time series of fluctuating frequencies sequenced by closeness. Each strand will have a start and end and may contain zero values in its time series where the frequency dropped out due to a bifurcation.

### Mean Intermittent frequency correlation

For each pair of oscillators, *m* and *n*, there is a collection of fluctuating frequency strands scattered over the frequency domain and stretching over time. We correlate each strand in oscillator *m* with each strand in oscillator *n*, for all oscillator combinations in the network. We do this by passing a 100 millisecond window over time in incremental steps of 1 millisecond. In each of these windows we take the time series data in that window for all pairs of strands *i* and *j*, where *i* and *j* are from different oscillators *m* and *n* respectively. For each pair of windowed strands we then remove time points from each strand where both strands do not have a response at that time point in the window, or when both the frequencies in the strands at one time point are the same as at the previous time point. This results in two time series strands for the window at time *t*, *w_m,i_(t)* and w*_n,j_ (t).* Both are the same length and are potentially shorter than the window size. Each time series contains only data where both original strands have a frequency response and they are both fluctuating. We then correlate these two series. We select only correlations where the coefficient is greater than or equal to 0.5 or the coefficient of the anti-correlation is less than or equal to −0.5, and the p-value for either is less than 0.05. By randomizing the order of one of the series and performing the same correlation and selection process we obtain a phantom correlation. We use phantom correlations to evaluate the importance of the measure of real correlation found. For both types of correlation we calculate the *mean intermittent frequency correlation* as follow: 
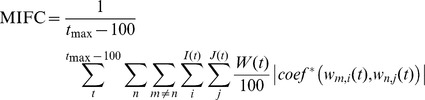



Where *n* and *m* are oscillators, *I(t)* and *J(t)* are the total number of strands in the window at time *t* for each oscillator respectively, and *i* and *j* are particular strands within each oscillator. coef^*^ defines the value of a significant correlation coefficient as previously described. *W(t)* is the length of the two series *w_m,i_(t)* and *w_n,j_ (t)*, that only contain time points that have a fluctuating frequency response in both original windowed strands. 100 is the length of the window. Thus the significant correlation coefficient is normalized according to the length of the two series in that window. *t* is the time of the particular window and *t_max_* is the length of the simulation time. The metric calculates all pairwise significant frequency correlations between all oscillators, normalizes them by their length, and averages them over time.

### Synchronisation metric

All the simulations in this work consisted of 10 neural oscillators connected together. Each neural oscillator consisted of an excitatory layer and an inhibitory layer. We only calculated synchrony for the excitatory neuron layers in the oscillators. The spikes of each neuron in each excitatory layer were binned over time, and then a Gaussian smoothing filter was passed over the binned data to produce a continuous time varying signal. Following this, we performed a Hilbert transform on the mean-centred filtered signal in order to identify its phase. No band-pass filtering was performed during this process. The synchrony at time *t* was then calculated as follows:
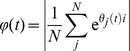


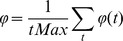
where *θ_j_(t)* is the phase at time *t* of oscillatory population *j*. *i* is the square root of -1. *N* is the number of oscillators, and *t_max_* is the length of time of the simulation.

### Coalition entropy

Coalition entropy measures the variety of metastable states entered by a system of oscillators. We only calculated coalition entropy for the excitatory neuron layers in the oscillators. As with the synchrony metric, we calculated the phase of each oscillator at each time point *t* using a Hilbert transform. We then performed clustering at each time point by picking the two most synchronous oscillators/coalitions using the first equation defined for the synchrony metric. Once a pair was identified they were joined to form a new coalition and the new coalitions mean complex exponential phase was calculated for use in the future most synchronous pair selection process. A threshold of 0.05 from full synchrony was used to limit the cluster merging. The process was repeated until no oscillators/coalitions fell within the threshold to allow merging into a new coalition.

Having identified the synchronous coalitions at each point in time we calculated the probability *p(s)* of each coalition occurring from the number of times it appeared throughout the simulation. The coalition entropy *H_c_* was then calculated as follows:
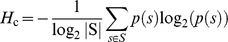
where |S| is the number of possible coalitions given the number of oscillators in the system.

### Hardware acceleration

Each of our simulations required 10 neural PING nodes each of 250 neurons, resulting in 2500 neurons and ≈880,000 synapses, and entailing an immense computational burden across the entire parameter space sweep in our experiments. To cope with this, we used the NeMo neural network simulator, which processes neurons concurrently on general purpose graphics processing units (GPUs) [43]. The NeMo software permits the addition of user plugins for neural models, which allowed us to implement both QIF and HH models for the NeMo simulator facilitating the work presented here.

## Results

**Figure 3:**
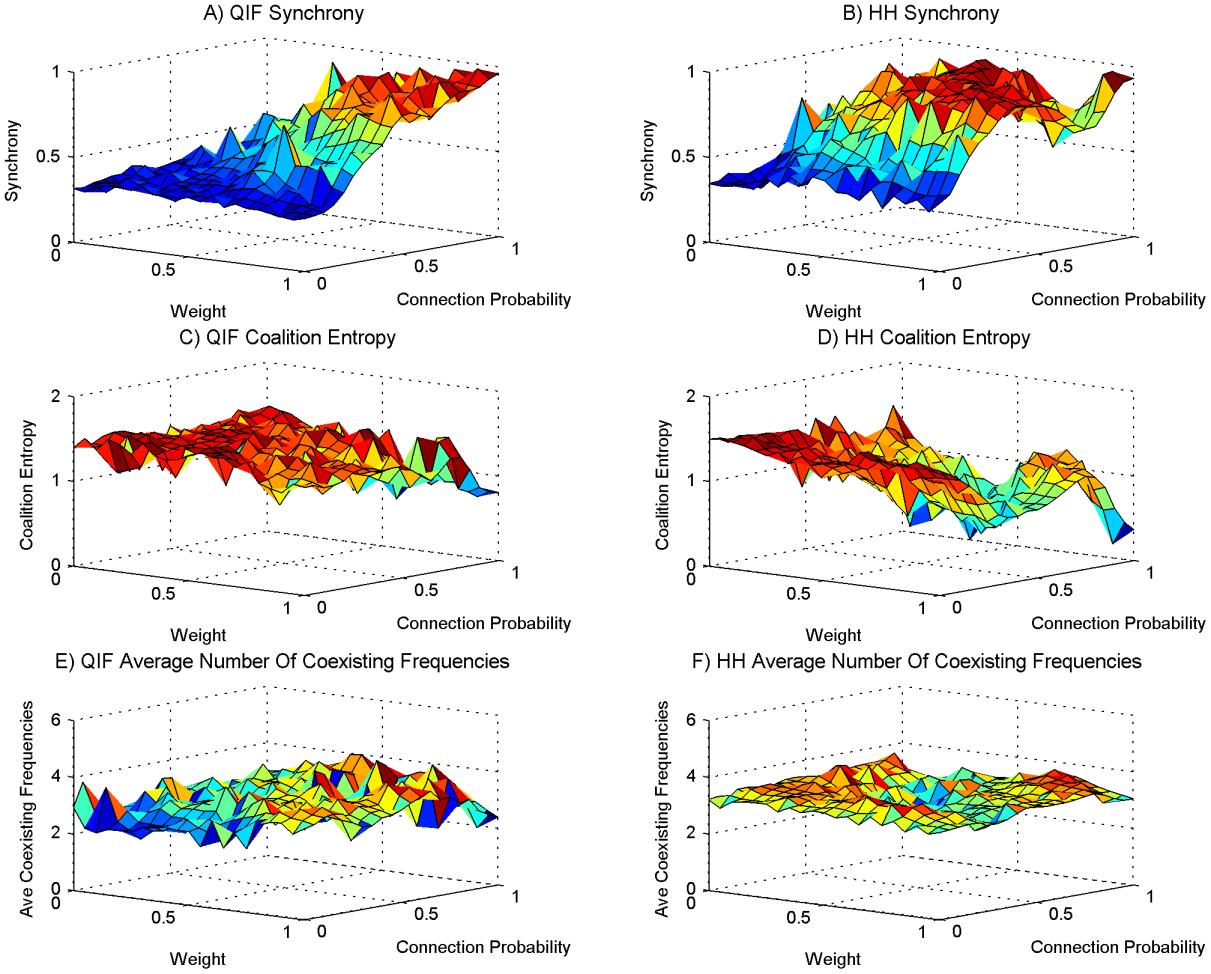
Synchrony, coalition entropy, and the number of coexisting frequencies. Each simulation uses 10 neural PING oscillator nodes with the connection probability and weight being the same between all nodes on a single simulation run. Each separate simulation uses a different connection probability and weight drawn from a uniform distribution between 0 and 1. (A) The overall synchrony in the networks using the QIF neuron model, (B) same as panel A for the HH neuron model. (C) The coalition entropy in the networks using the QIF neuron model, (D) same as panel C for the HH neuron model. (E) The average number of coexisting frequencies per oscillator at each time point in the networks using the QIF neuron model, (F) the same as panel E for the HH neuron model.

**Figure 4:**
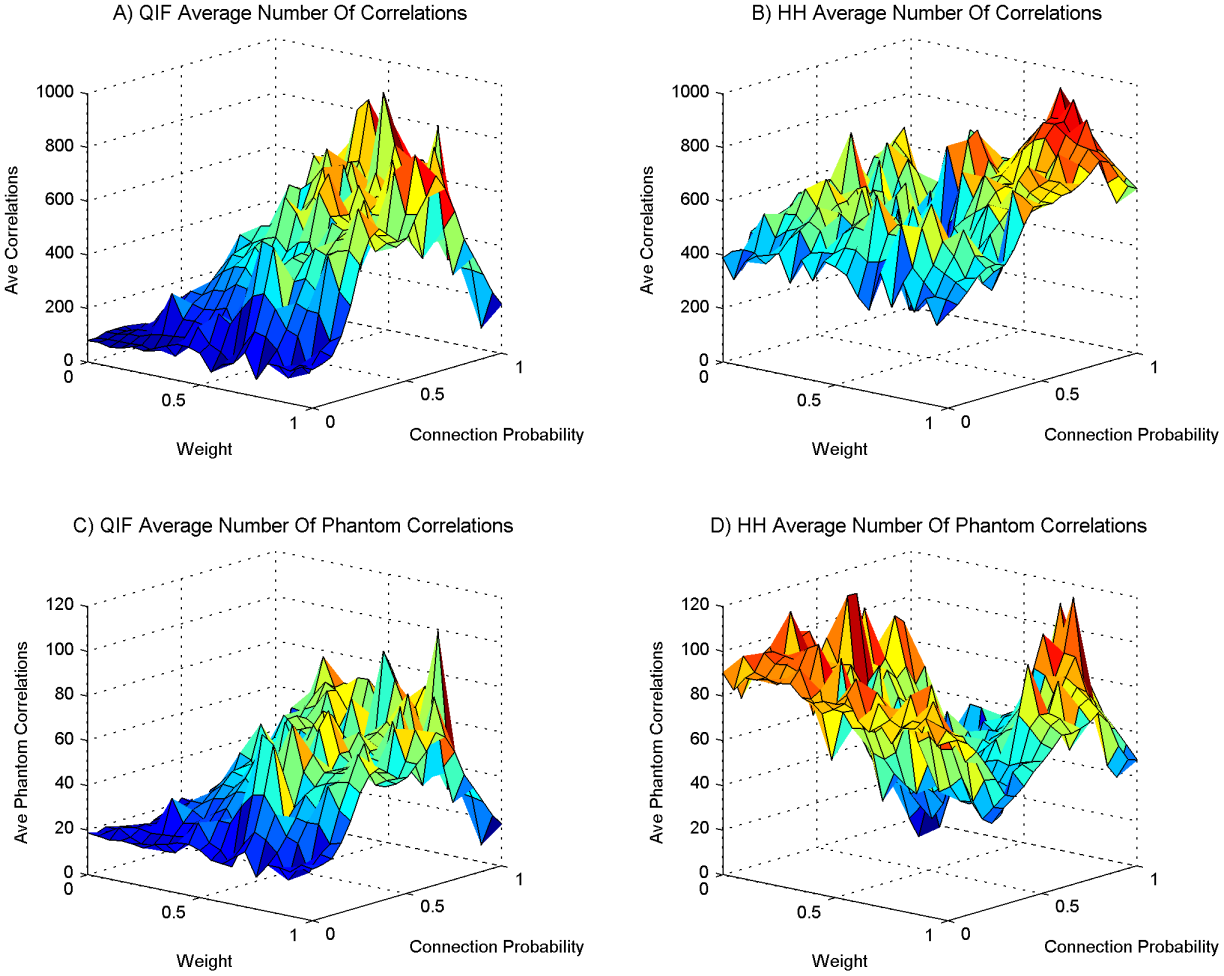
The number of correlations found. The setup is the same as for figure 3. (A) The average number of mean intermittent frequency correlations found for networks using the QIF neuron model, (B) same as panel A for the HH neuron model. (C) The average number of phantom mean intermittent frequency correlations found for networks using the QIF neuron model, (D) the same as panel C for the HH neuron model.

**Figure 5:**
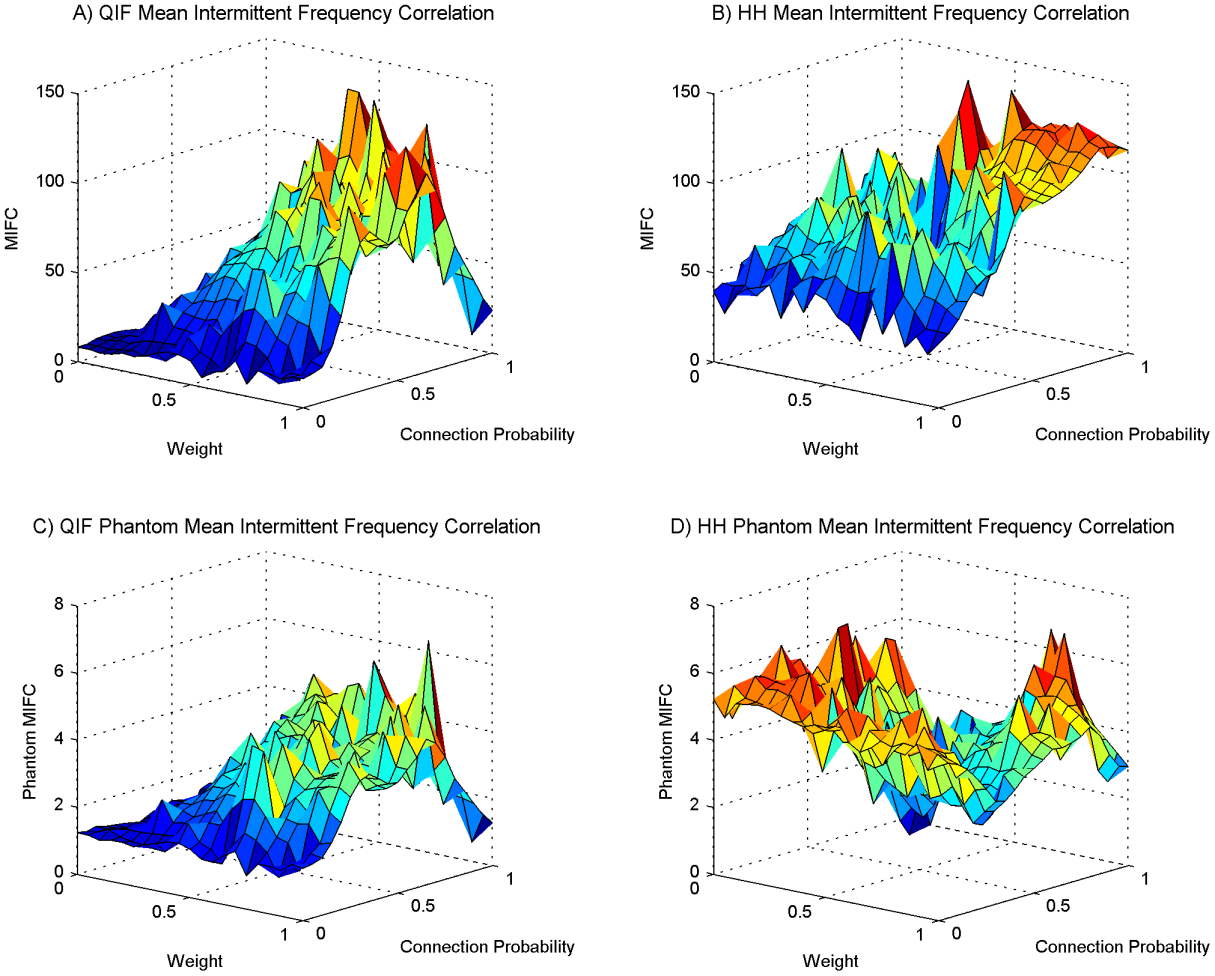
Mean intermittent frequency correlation. The setup is the same as for figure 3 and subsequent figures. (A) The mean intermittent frequency correlation for networks using the QIF neuron model, (B) the same as panel A for the HH neuron model. (C) The phantom mean intermittent frequency correlation for networks using the QIF neuron model, (D) the same as panel C for the HH neuron model. The mean intermittent frequency metric selects correlations where the coefficient > = 0.5 and p < = 0.05, and all correlations are normalised by the length of the time series strands.

**Figure 6:**
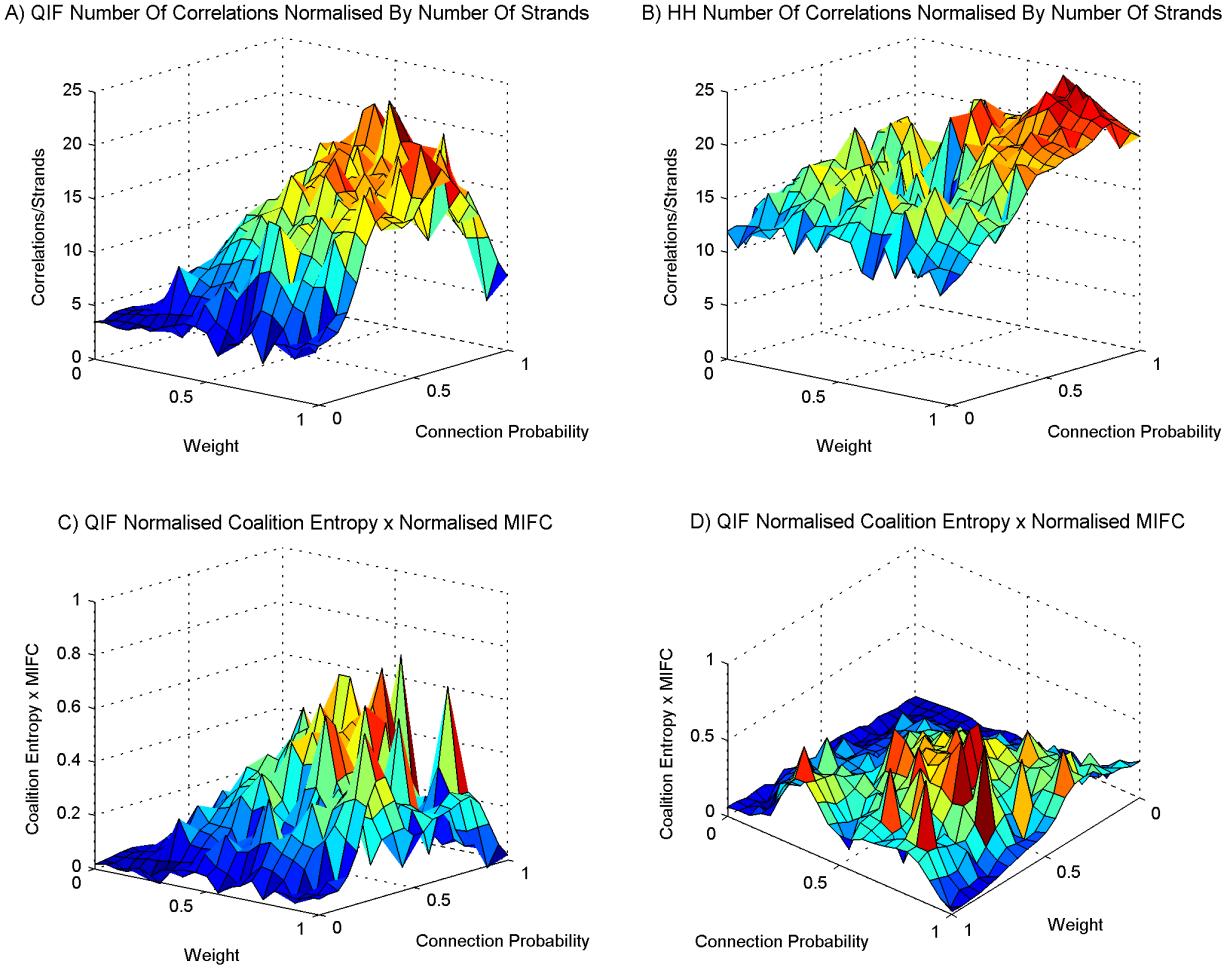
Average number of correlations, and the peak of modulated exploration. The setup is the same as for figure 3 and subsequent figures. (A) The average number of mean intermittent frequency correlations found for networks using the QIF neuron model, (B) the same as panel A for the HH neuron model. The number of correlations found has been normalized by the number of coexisting frequency time series in all oscillators on each simulation run. The figure shows, on average, how many frequencies in other oscillators each individual frequency is interacting with at each time point. (C) and (D) show from two different angles a combination of mean intermittent frequency correlation and coalition entropy for the QIF neuron model. The values of both metrics have been normalised before multiplying them together. The graphs emphasise a peak area, and in this area there is also a linear relationship between weight and connection probability. This peak area facilitates modulated exploration of a large repertoire of different coalitions.

We performed a series of experimental simulations in each of which 10 neural PING oscillators were chosen from the set we had evolved with intrinsic frequencies ranging from 30 Hz to 50 Hz. The probability of one oscillator providing neural input to another was determined with a given probability *P*. The probability *P* was the same for all oscillator to oscillator connections in the same experimental simulation. Given that a connection was established from oscillator *n* to oscillator *m* the excitatory neurons in oscillator *n* would form synaptic connections to the excitatory neurons in oscillator *m*. The number of synaptic connections formed was 20 percent of the 40000 possible synaptic connections from the 200 excitatory neurons in oscillator *n* to the 200 excitatory neurons in oscillator *m*. For all synaptic connections formed the weight of the synapse was set to *W*. The value for *W* and *P* were randomly chosen at the beginning of each experimental simulation from a uniform distribution between 0 and 1. 250 simulations were performed for the QIF neural model and 250 simulations for the HH neural model. As the weight and connection probability for each simulation were chosen at random these data points are scattered throughout the space, the 250 simulations thus constitute a scattered sweep of weight and the inter-oscillator network connection sparsity. Figures 3, 4, 5 and 6 show various measures taken from these 250 simulations of QIF and HH neuron models. These are analysed and discussed in detail below. In each of these figures a surface has been fitted to the underlying trend of the 250 data point for each measure depicted. The original scatter plots for the 250 data points of each measure are included in the supporting information (figures S1, S2, S4, S4 respectively).

Throughout each simulation external stimulus input was provided to each neural oscillator from a Poisson process with parameter λ = 4.375. For QIF models the inputs were scaled by 8 and for the HH models the inputs were scaled by 15 in order to provide sufficient stimulus to induce firing. Each experiment was run for 2000 ms of simulated time. After each experiment, the firing activity of the excitatory layers in each oscillator was used to calculate synchrony, coalition entropy and the mean intermittent frequency correlation as described in the Methods section. The first 500 ms of each simulation were discarded in the calculation of these metrics to eliminate initial transients.

Figures 3a and 3b show the synchrony through the sweep for QIF and HH models respectively. Unsurprisingly, and in accord with previous findings [21], synchrony increases as connectivity increases. The neural systems never reach full synchrony as at their maximum value these systems exhibit deviations from full synchrony. The value of 0.25 synchrony in the area of low weight and low connection probability represents no synchrony at all except coincidental alignments in phase. The HH model shows a dip in synchrony around the area of maximum weight and maximum connection probability. This is due to “saturation”, by which we mean all the neurons in the oscillators in the network often fired continuously rather that oscillating. This phenomenon appears as an artefact in the same weight and connection probability area for all measures presented for the HH model.

Figures 3c and 3d show the coalition entropy through the sweep for QIF and HH models respectively. The trend for coalition entropy takes the reverse form to synchrony, decreasing as synchrony increases, and the oscillators become more aligned in phase for more of the time. The measure of coalition entropy used cannot distinguish coincidentally synchronous coalitions from those that are genuinely coupled. However, when we contrast with the graphs of synchrony we can get an idea of what is happening. Regions of the parameter space with low weight and low connection probability exhibit high coalition entropy, but the same regions present low values for synchrony. This suggests that the many coalitions that appear are constituted by very short coincidental alignments in phase that are not capable of significant information transfer [17]. The mid parameter space area shows fairly high values for synchrony, indicating the capacity for substantial information transfer, as well as high coalition entropy indicating transfer between many different groups at different times. The region of the parameter space in which the weight and connection ratio are high facilitates more information transfer but less variation in coalitions.

Figures 3e and 3f show the average number of coexisting frequencies in a single oscillator at any one time in each simulation for QIF and HH models. Interestingly for the QIF model, as the causal influences increase through stronger weights and greater connectivity, the number of coexisting frequencies rises. This indicates that stronger causal interactions between neural populations, that otherwise oscillate at a single intrinsic frequency, are a source of increased spectral complexity. The HH model shows a dip in the mid area of the parameter space, after which the number of coexisting frequencies rises. Whilst this latter area also demonstrates that causal interactions increase spectral complexity in areas where there are stronger inter-oscillator influences, it is interesting to note that in the area of weaker influences this model also generates a large number of coexisting frequencies. This latter phenomenon will be elucidated later.

Taking a look at the number of significant correlations found through the sweep, figures 4a and 4b show that both QIF and HH models display an increased number of correlations as the synaptic weight and connectivity increases, although the HH model has a less pronounced incline. The increase has a similar trend to that of synchrony. The data indicate that correlated fluctuations in frequency imply more episodes of synchrony, suggesting that the fluctuating influences between oscillators are moving each other towards synchronous behaviour. As the number of significant correlations is so high, we can conclude that this influence works across frequency bands. To control for coincidental (“phantom”) correlations, we contrast the number of significant correlations found to the number found when reversing one of the time series before correlating (figures 4c and 4d). We see that for the QIF model, although many significant phantom correlations are found and they follow the same trend, the number found is an order of magnitude less than the number of real correlations. The HH model displays a similar ratio between real and phantom correlations. However, the region of low weight and connection probability shows large numbers of correlations in line with the greater number of coexisting frequencies found in that area in figure 3f.

The mean intermittent frequency correlations are shown in figures 5a and 5b for QIF and HH models respectively. As causal influence between oscillators increases in the network this correlation measure increases, meaning that correlation directly reflects causation in this case. Data not presented show that when separating these data into positive correlations and anti-correlations both follow the same trend (this data separation is included in figure S5 in the supporting information). The metric not only identifies the significant correlations but also normalizes each of these correlations by the length of the intermittent fluctuating frequency time series. The resulting values are therefore always much less than the number of correlations. The mean intermittent frequency correlation shown for phantom correlations in figures 5c and 5d is very low, peaking at around 7 compared to real correlations, which peak at around 140. The ratio is double that found for the simple “number of significant correlations found” of figure 4 and so is even stronger justification that the correlations found are significant. For the HH model, the area of low weight and connection probability shows small mean intermittent frequency correlation values, in contrast with the corresponding ‘number of correlations found’ shown in figure 4b due to greater number of coexisting frequencies seen in figure 3f. This is because, although significant correlations are found, they are being normalized to a lesser value by length, meaning that these are very short time series. This is further reason for discarding high coalition entropy values in this area, on the grounds that they are not due to any consequential interactions but are merely coincidental. The performance of HH networks around this low parameter region is therefore very erratic compared to the behaviour in the mid and high connectivity regions which exhibits stable and modulatory influences.

In figures 6a and 6b, the number of correlations found has been normalized by the number of coexisting frequency time series in all oscillators on each run. These figures show, on average, how many frequencies in other oscillators each individual frequency is interacting with at each time point. The mid area of figures 3e and 3f show ≈3 coexisting time series per oscillator at each time point. The mid and high parameter region in figures 6a and 6b show many more than the 9 interactions we would expect if each frequency was only interacting with frequencies in other oscillators that are in the same frequency band. We can safely conclude from this that frequencies in different neural populations communicate across bands. This type of complexity is not manifest in simple oscillator models, a shortcoming that is most evident at high levels of synchrony when simple oscillator models, unlike systems of neurons, display only a single shared frequency.

Figures 6c and 6d show, from two angles, a combination of QIF mean intermittent frequency correlation in figure 5a with the QIF coalition entropy in figure 3d. To obtain this combination we normalized the mean intermittent frequency correlation between 0 and 1, and the coalition entropy between −1 and 1, and multiplied the results together. The coalition entropy was normalized between −1 and 1 to emphasize the dominant trend in the graphs which lies in the upper half. There is a vector from weight value 0.35 and connection probability 1 to weight value 1 and connection probability 0.35 at which the amount of correlation between fluctuating frequencies across oscillators coincides with a measure of coalition entropy such that they are at a combined peak. This area, in which the two metrics are balanced, facilitates metastable dynamics in which there is a richness of influence and interaction between different oscillators and across frequency bands modulating each other's behaviour, enabling the exploration of a large repertoire of different coalitions. It is noteworthy that there is a linear relationship between weight and connection probability at which this is best facilitated. The medium-to-high level of synchrony in this area further suggests that the conditions for information transfer between populations are fulfilled [17]. These traits are desirable in order to facilitate a system versatile at exploration, integration and communication of functionally related areas during cognitive processing [44], [45], [46].

## Discussion

A general rule of thumb for oscillator systems is that greater connectivity produces more synchrony. Unlike simple oscillator models, systems of spiking neurons display greater spectral complexity with many coexisting frequencies existing in a single oscillator at one time. The work presented here demonstrates that this complexity increases with connectivity, not only in the number of coexisting frequencies but also in the amount of interaction across frequency bands. As causal interactions increase, so does correlation between these fluctuating frequencies, as well as the tendency towards more and longer episodes of synchrony and information transfer, which implies that they are modulating each other towards communication.

Our particular interest lies in theories of metastability in which neural behaviour produces episodes of synchronization and desynchronization between oscillating populations, for which the combined effect amongst a collection of oscillators is to explore many different coalitions over time. The results presented in this paper identify an area in the weight and connectivity space at which spiking neuron models are at a balance in which coalition entropy is exhibited due to the influential modulation between populations through their oscillatory behaviour. In this area of balance the neural systems influence each other across frequency bands in a way that promotes exploration of, and communication between, coalitions. This is because the fluctuating oscillatory frequencies in each neural population modulate each other so as to drive the system towards episodes of synchrony between different neural populations, enabling communication between them. Whilst doing this, the variation in synchronous coalitions of neural populations over time is kept very high, and hence this area of the connectivity space may be described as encouraging exploration. We suggest that a dynamical system whose component parts interact so as to direct the system through varieties of coalitions would form a good basis for contextual exploration as well as integration among, and communication between functionally related areas during cognitive processing. Further to this, maintaining large repertoire of synchronous coalitions promotes versatile exploration of novel functional combinations, a desirable trait when problem solving.

Athough the present work is intended primarily as a computational and theoretical study, preliminary empirical work has provided evidence for comparable metastable dynamics in the resting state human brain [47]. The present work is a step towards better understanding of how the combined activity of individual spiking neurons gives rise to the of formation of coherent oscillating assemblies, and how the dynamics between these assemblies evolve over time. Our longer term aim is to work towards elucidating such phenomena both in the brains of living organisms and in the architecture of computational models, phenomena that permit the exploration, integration and communication of functionally related neural areas during cognitive processing.

## Supporting Information

Figure S1 **Synchrony, coalition entropy, and the number of coexisting frequencies scatter plot.** This plot is of the original 250 data points from which the surface plot of figure 3 was created. The setup is the same as for figure 3 and subsequent figures. Each simulation uses 10 neural PING oscillator nodes with the connection probability and weight being the same between all nodes on a single simulation run. Each separate simulation uses a different connection probability and weight drawn from a uniform distribution between 0 and 1. (A) The overall synchrony in the networks using the QIF neuron model, (B) same as panel A for the HH neuron model. (C) The coalition entropy in the networks using the QIF neuron model, (D) same as panel C for the HH neuron model. (E) The average number of coexisting frequencies per oscillator at each time point in the networks using the QIF neuron model, (F) the same as panel E for the HH neuron model.
(TIF)

Figure S2 **The number of correlations found scatter plot.** This plot is of the original 250 data points from which the surface plot of figure 4 was created. The setup is the same as for figure 3 and subsequent figures. (A) The average number of mean intermittent frequency correlations found for networks using the QIF neuron model, (B) same as panel A for the HH neuron model. (C) The average number of phantom mean intermittent frequency correlations found for networks using the QIF neuron model, (D) the same as panel C for the HH neuron model.
(TIF)

Figure S3 **Mean intermittent frequency correlation scatter plot.** This plot is of the original 250 data points from which the surface plot of figure 5 was created. The setup is the same as for figure 3 and subsequent figures. (A) The mean intermittent frequency correlation for networks using the QIF neuron model, (B) the same as panel A for the HH neuron model. (C) The phantom mean intermittent frequency correlation for networks using the QIF neuron model, (D) the same as panel C for the HH neuron model. The mean intermittent frequency metric selects correlations where the coefficient > = 0.5 and p < = 0.05, and all correlations are normalised by the length of the time series strands.
(TIF)

Figure S4 **Average number of correlations, and the peak of modulated exploration scatter plot.** This plot is of the original 250 data points from which the surface plot of figure 6 was created. The setup is the same as for figure 3 and subsequent figures. (A) The average number of mean intermittent frequency correlations found normalised by the number of coexisting strands for networks using the QIF neuron model, (B) the same as panel A for the HH neuron model. The number of correlations found has been normalised by the number of coexisting frequency time series in all oscillators on each simulation run. The figure shows, on average, how many frequencies in other oscillators each individual frequency is interacting with at each time point. (C) and (D) show from two different angles a combination of mean intermittent frequency correlation and coalition entropy for the QIF neuron model. The values of both metrics have been normalised before multiplying them together. The graphs emphasise a peak area, and in this area there is also a linear relationship between weight and connection probability. This peak area facilitates modulated exploration of a large repertoire of different coalitions.
(TIF)

Figure S5 **Separation of positive and anti mean intermittent frequency correlation.** (A) The positive mean intermittent frequency correlation for the QIF neuron model. (B) The same as panel A for the HH neuron model. (C) The anti mean intermittent frequency correlation for the QIF neuron model. (B) The same as panel C for the HH neuron model.
(TIF)

File S1 Supporting Figure Descriptions.
DOC)
