# Supplementary figures and images for: Metastability and Inter-Band Frequency Modulation in Networks of Oscillating Spiking Neuron Populations
Source: PLoS One. 2013 Apr 16;8(4):e62234. doi: 10.1371/journal.pone.0062234 (PMC3628585; doi:10.1371/journal.pone.0062234)

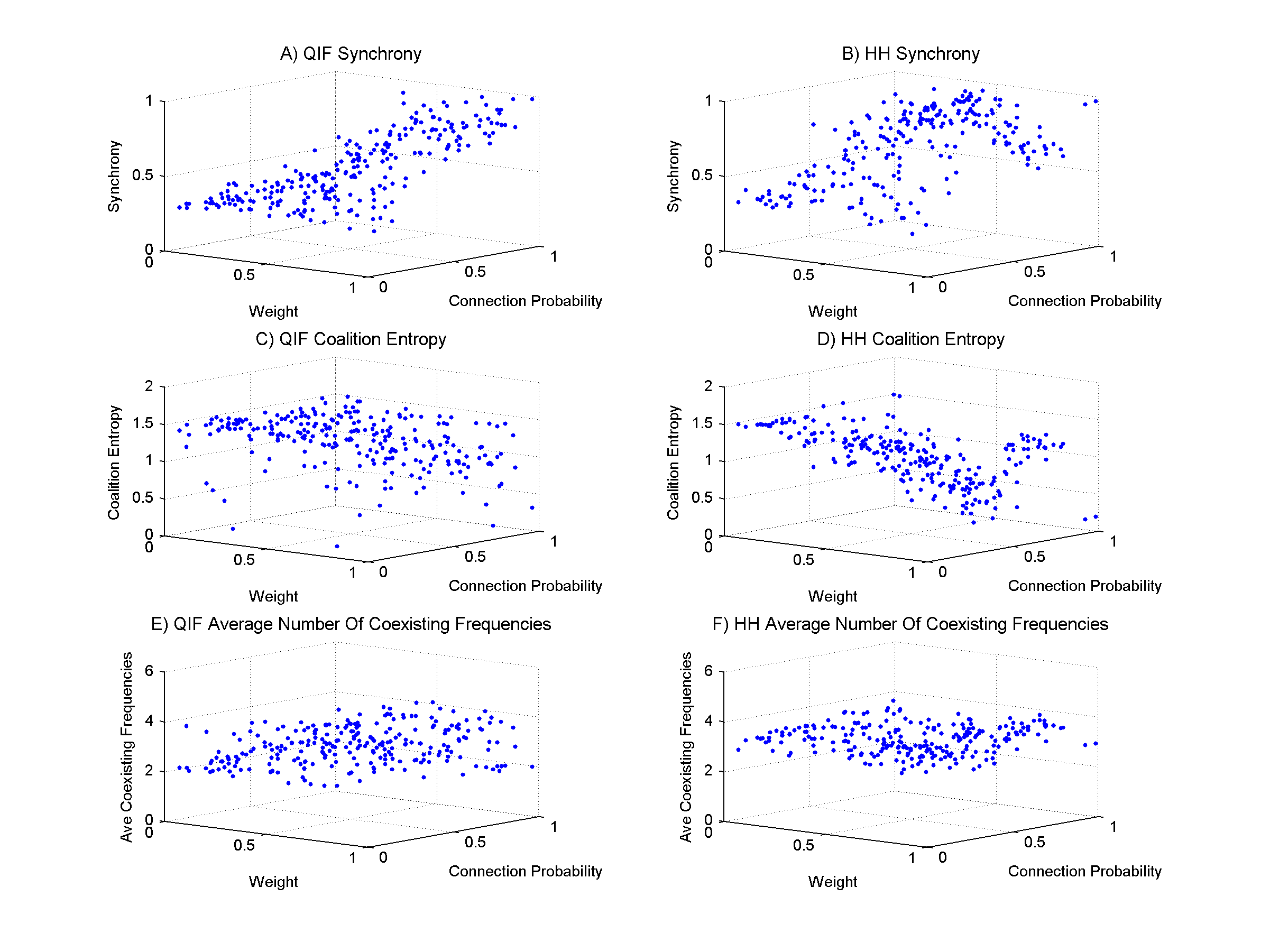

Supplement: Figure S1 — Synchrony, coalition entropy, and the number of coexisting frequencies scatter plot. This plot is of the original 250 data points from which the surface plot of figure 3 was created. The setup is the same as for figure 3 and subsequent figures. Each simulation uses 10 neural PING oscillator nodes with the connection probability and weight being the same between all nodes on a single simulation run. Each separate simulation uses a different connection probability and weight drawn from a uniform distribution between 0 and 1. (A) The overall synchrony in the networks using the QIF neuron model, (B) same as panel A for the HH neuron model. (C) The coalition entropy in the networks using the QIF neuron model, (D) same as panel C for the HH neuron model. (E) The average number of coexisting frequencies per oscillator at each time point in the networks using the QIF neuron model, (F) the same as panel E for the HH neuron model. (TIF) [file pone.0062234.s001.tif]

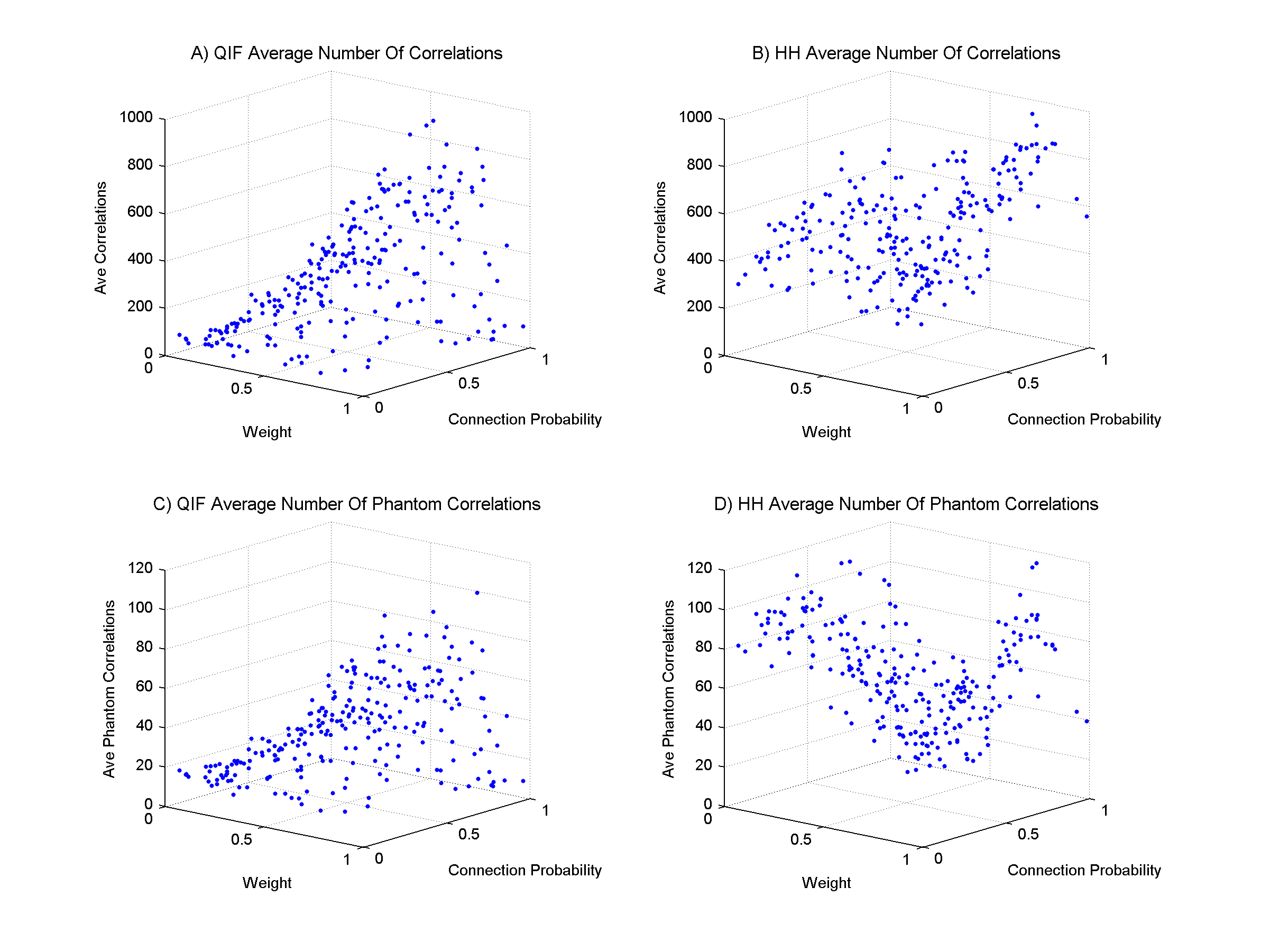

Supplement: Figure S2 — The number of correlations found scatter plot. This plot is of the original 250 data points from which the surface plot of figure 4 was created. The setup is the same as for figure 3 and subsequent figures. (A) The average number of mean intermittent frequency correlations found for networks using the QIF neuron model, (B) same as panel A for the HH neuron model. (C) The average number of phantom mean intermittent frequency correlations found for networks using the QIF neuron model, (D) the same as panel C for the HH neuron model. (TIF) [file pone.0062234.s002.tif]

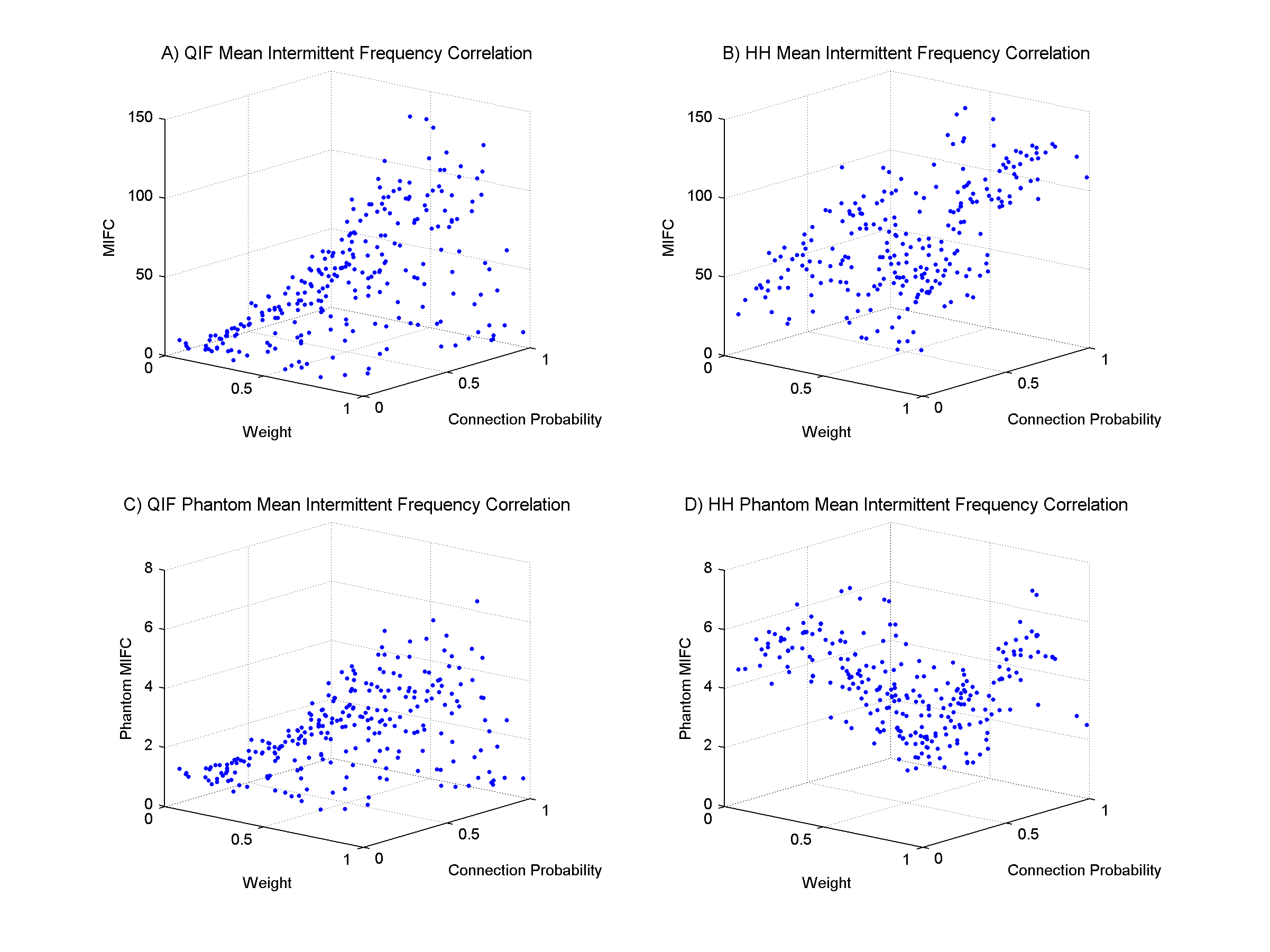

Supplement: Figure S3 — Mean intermittent frequency correlation scatter plot. This plot is of the original 250 data points from which the surface plot of figure 5 was created. The setup is the same as for figure 3 and subsequent figures. (A) The mean intermittent frequency correlation for networks using the QIF neuron model, (B) the same as panel A for the HH neuron model. (C) The phantom mean intermittent frequency correlation for networks using the QIF neuron model, (D) the same as panel C for the HH neuron model. The mean intermittent frequency metric selects correlations where the coefficient > = 0.5 and p < = 0.05, and all correlations are normalised by the length of the time series strands. (TIF) [file pone.0062234.s003.tif]

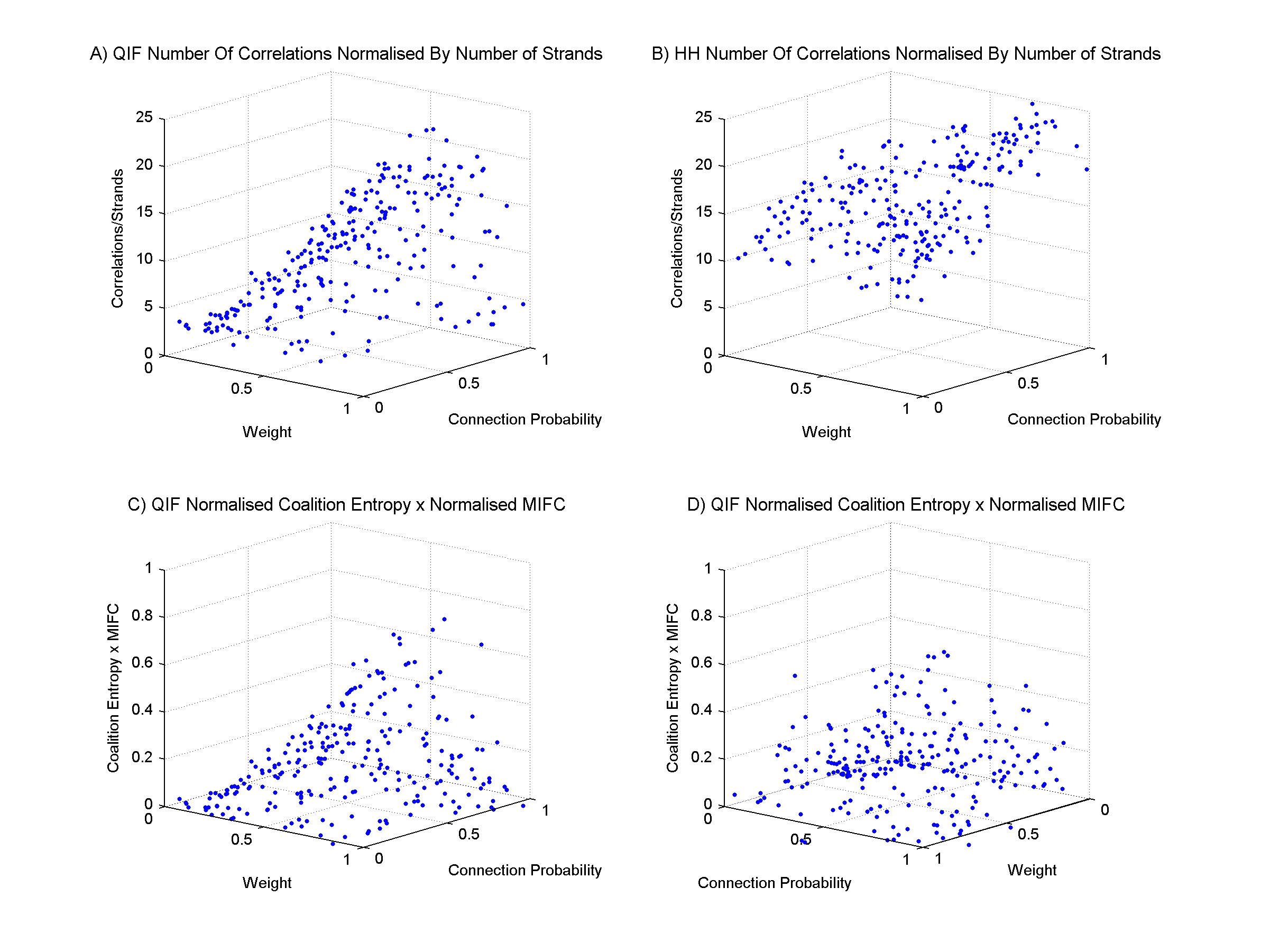

Supplement: Figure S4 — Average number of correlations, and the peak of modulated exploration scatter plot. This plot is of the original 250 data points from which the surface plot of figure 6 was created. The setup is the same as for figure 3 and subsequent figures. (A) The average number of mean intermittent frequency correlations found normalised by the number of coexisting strands for networks using the QIF neuron model, (B) the same as panel A for the HH neuron model. The number of correlations found has been normalised by the number of coexisting frequency time series in all oscillators on each simulation run. The figure shows, on average, how many frequencies in other oscillators each individual frequency is interacting with at each time point. (C) and (D) show from two different angles a combination of mean intermittent frequency correlation and coalition entropy for the QIF neuron model. The values of both metrics have been normalised before multiplying them together. The graphs emphasise a peak area, and in this area there is also a linear relationship between weight and connection probability. This peak area facilitates modulated exploration of a large repertoire of different coalitions. (TIF) [file pone.0062234.s004.tif]

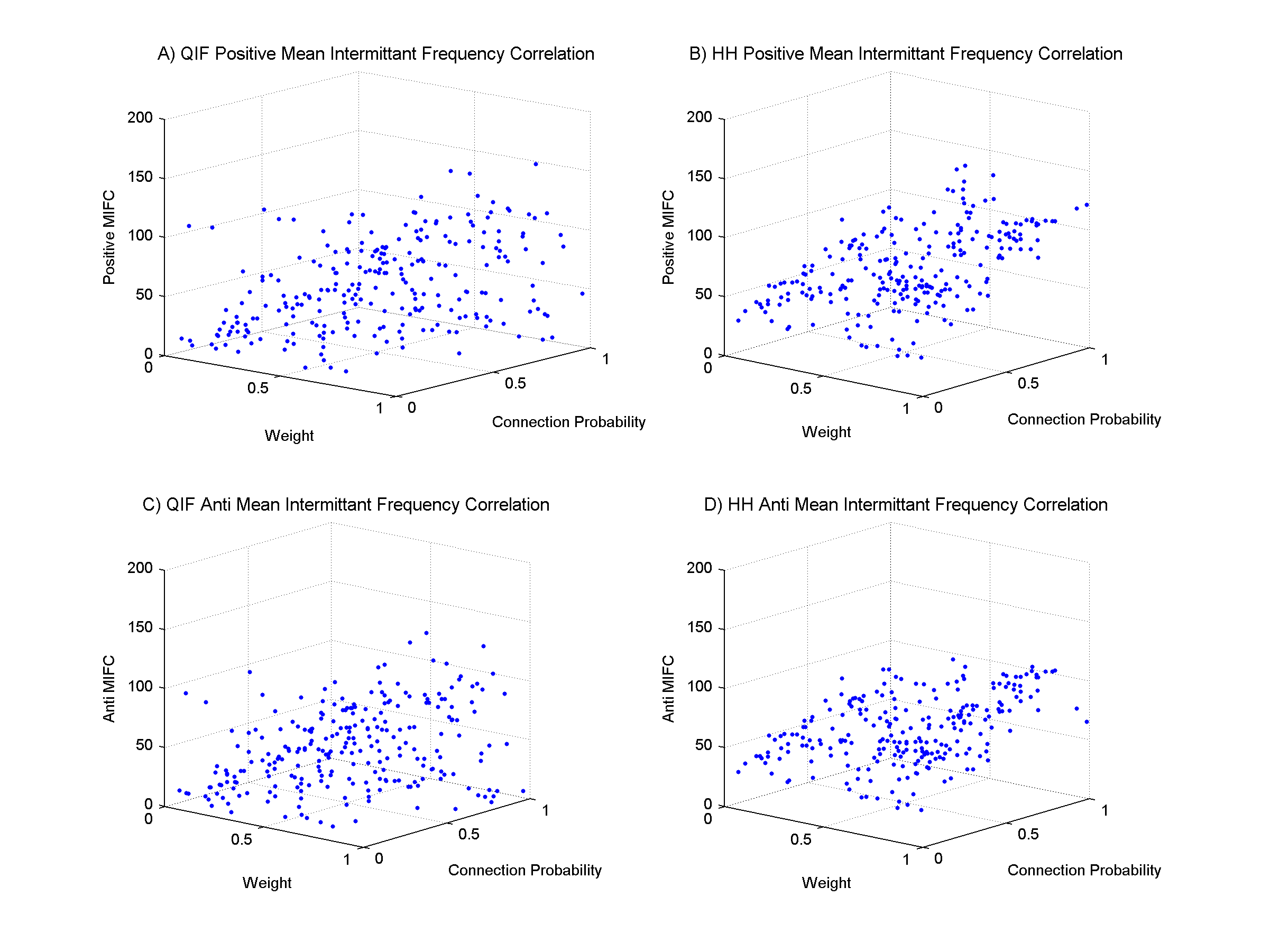

Supplement: Figure S5 — Separation of positive and anti mean intermittent frequency correlation. (A) The positive mean intermittent frequency correlation for the QIF neuron model. (B) The same as panel A for the HH neuron model. (C) The anti mean intermittent frequency correlation for the QIF neuron model. (B) The same as panel C for the HH neuron model. (TIF) [file pone.0062234.s005.tif]
