# Supplementary material for: Metastability and Inter-Band Frequency Modulation in Networks of Oscillating Spiking Neuron Populations
Source: PLoS One. 2013 Apr 16;8(4):e62234. doi: 10.1371/journal.pone.0062234 (PMC3628585; doi:10.1371/journal.pone.0062234)
Supplement: File S1 — Supporting Figure Descriptions. DOC) [file pone.0062234.s006.doc]

## Supporting Information

In the main paper, figures 3 to 6 show various measures taken from the 250 simulations for each of the two neuron models. In each of these figures a surface has been fitted to the underlying trend of the 250 data point for each measure depicted. Figures S1 to S4 are scatter plots of the 250 data points, of the same measures, and from which the underlying trend was extracted and later used in the surface plots of figures 3 to 6.

In the paper we state*: “The mean intermittent frequency correlations are shown in figures 5a and 5b for QIF and HH models respectively. As causal influence between oscillators increases in the network this correlation measure increases, meaning that correlation directly reflects causation in this case. Data not presented shows that when separating these data into positive correlations and anti-correlations both follow the same trend.”* Figure S5 shows this separation data not included in the main paper.
